# Palaeoneurological clues to the evolution of defining mammalian soft tissue traits

**DOI:** 10.1038/srep25604

**Published:** 2016-05-09

**Authors:** J. Benoit, P. R. Manger, B. S. Rubidge

**Affiliations:** 1 Evolutionary Studies Institute (ESI), University of the Witwatersrand, PO Wits, 2050, Johannesburg, South Africa; 2 School of Anatomical Sciences, University of the Witwatersrand, 7 York Road, Parktown, 2193, Johannesburg, South Africa; 3 School for Geosciences, University of the Witwatersrand, PO Wits, 2050, Johannesburg, South Africa

## Abstract

A rich fossil record chronicles the distant origins of mammals, but the evolution of defining soft tissue characters of extant mammals, such as mammary glands and hairs is difficult to interpret because soft tissue does not readily fossilize. As many soft tissue features are derived from dermic structures, their evolution is linked to that of the nervous syutem, and palaeoneurology offers opportunities to find bony correlates of these soft tissue features. Here, a CT scan study of 29 fossil skulls shows that non-mammaliaform Prozostrodontia display a retracted, fully ossified, and non-ramified infraorbital canal for the infraorbital nerve, unlike more basal therapsids. The presence of a true infraorbital canal in Prozostrodontia suggests that a motile rhinarium and maxillary vibrissae were present. Also the complete ossification of the parietal fontanelle (resulting in the loss of the parietal foramen) and the development of the cerebellum in Probainognathia may be pleiotropically linked to the appearance of mammary glands and having body hair coverage since these traits are all controlled by the same homeogene, Msx2, in mice. These suggest that defining soft tissue characters of mammals were already present in their forerunners some 240 to 246 mya.

Understanding the origin and evolution of mammary glands and hairs in mammals is one of the most fascinating and intensive fields of biological research (e.g.^1^, reviewed in^2^,^3^). Both features are signature traits of extant mammals and their evolution is tightly intermeshed genetically, functionally, and morphologically, as modern lactation might have evolved from a specialization of hairs and dermal glands as exemplified in the monotremes^2^,^3^,^4^. Molecular evidence point out that hair and mammary glands might be older than 200 Ma^3^. As hair and mammary glands are soft tissue, they are not readily fossilized. A fossil record for mammary glands does not exist^2^, and the oldest known fossilized hairs date to the Late Jurassic^5^. Hair-like structures discovered inside coprolites dating from the Late Permian suggest that fur coverage might have existed as early as 252 to 254 mya, provided that these structures are not artifacts, remains of plants, worms or fungal material^6^,^7^.

Facial and body tactile sensibility is often directly related to pilosity, and given that stem mammaliaforms and a number of non-mammaliaform therapsids (including cynodonts) were apparently nocturnal and may not have strongly relied on vision to monitor their environment^8^,^9^,^10^, the evolution of hair must have dramatically influenced the survival of the lineage leading to mammaliaforms, i.e. the Late Paleozoic and early Mesozoic non-mammaliaform therapsids commonly known as the ‘mammal-like reptiles’^11^,^12^. Because mammary glands and hairs are derived from dermic structures, their evolution is closely linked to that of the nervous system (including the central nervous system) and skin sensitivity^2^,^3^,^11^,^12^. Here we report the paleoneurology of a variety of non-mammaliaform therapsids (NMT) using *in silico* studies and X-ray microtomography (μCT). This study documents the evolution of these key soft tissue mammalian characters and demonstrates that both direct and indirect evidence for the evolution of such soft tissues can be addressed using the fossil record.

## Comparative description

**Figure 1:**
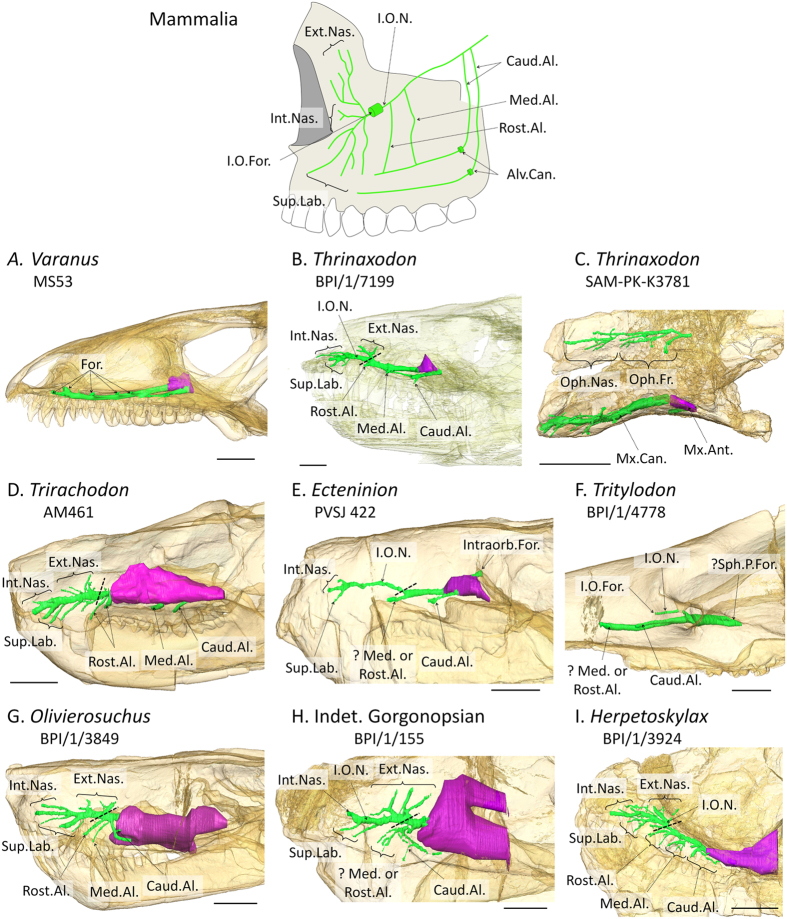
Lateral view, with the skull transparent, of the maxillary canal (in green) and maxillary antrum (in purple) in (**A**) the non-avian sauropsid *Varanus*, (**B**) the early cynodont *Thrinaxodon*, (**D**) the cynognathian *Trirachodon*, (**E**) the early probainognathian *Ecteninion*, (**F**) the probainognathian *Tritylodon*, (**G**) the therocephalian *Olivierosuchus*, (**H**) an indeterminated gorgonopsian, and (**I**) the biarmosuchian *Herpetoskylax*; and (**C**) the canal for the ophthalmic nerve in the early cynodont *Thrinaxodon* in dorsal view with the skull transparent. Rostral is to the left. Scale bar = 10 mm. The condition in *Homo* (top) illustrates the nomenclatural terms in Mammalia (after 20). Dotted line indicates the caudal extremity of the infraorbital nerve, which is marked by its bifurcation from the rostral alveolar nerve. The position of this dotted line is homologous to the infraorbital foramen in *Tritylodon* and mammaliaforms. Videos of the CT images of the specimens are available in the Supplementary Information. Abbreviations: Alv.Can., alveolar canal; Caud.Al., caudal alveolar rami; Ext.Nas., external nasal rami of the infraorbital nerve; For., foramina for the maxillary branch of the trigeminal nerve; Int.Nas., internal nasal rami of the infraorbital nerve; Intraorb.For., intraorbital foramen for the infraorbital nerve; I.O.For, infraorbital foramen; I.O.N., infraorbital nerve; Med.Al., medium alveolar nerve; Mx.Ant., maxillary antrum; Mx.Can., maxillary canal; Oph.Fr., frontal ramus of the ophtalmic nerve; Oph.Nas., nasal ramus of the ophtalmic nerve; Rost.Al., rostral alveolar nerve; Sph.P.For., sphenopalatine foramen; Sup.Lab., supralabial ramus of the infraorbital nerve.

The maxillary canal is a bony tube which runs parallel to the tooth row in the maxilla and premaxilla. In extant non-avian sauropsids, it carries the maxillary branch of the trigeminal nerve (CNV_2_), as well as a branch of the facial nerve (CNVII), and some blood vessels^13^,^14^,^15^. The maxillary canal communicates with tooth roots in therapsids^16^,^17^. As such, not all ramifications of the canal are relevant to the evolution of facial sensitivity and motility. In order to focus our study on the bony structures that carried tissues which actively played a role in the innervation, sensitivity, and nutrition of the face, only the parts of the canal that directly communicate with the external surface were segmented and are described herein. In accordance with the direct phylogenetic relationship unifying Mammaliaformes and NMT, and despite similarities with the anatomy of the maxillary canal in extant reptiles (Fig. 1A), the identification of the rami of the maxillary canal are based on the name of the corresponding ramus of the CNV_2_ in therian mammals. The aim is to maximize primary hypotheses of homology. However, the reader should keep in mind that these are bony structures that may have housed more than the CNV_2_. Comparisons with extant species are restricted to therian mammals as monotremes do not display the primitive condition for mammals since: (i) their rostrum (and incidentally the anatomy of their maxillary canal) is closely associated with their peculiar foraging strategies (i.e. aquatic invertebrate hunting for platypus and ant-eating for echidna); and (ii) in both the echidna and platypus the maxilla and mandible are covered with unique electroreceptors and mechanoreceptors innervated by an hypertrophied trigeminal nerve, leading to a dramatically derived condition^18^,^19^,^20^. As the maxillary canal is similar in most specimens examined, a detailed description is provided only for early cynodonts, the best documented taxon in our sample, and only diagnostic differences are listed for other taxa.

Early cynodonts display several small foramina for the external opening of the maxillary canal on their rostrum. The main axis of the maxillary canal can be divided into a caudal and a rostral part in early cynodonts. The caudal half extends from the maxillary antrum (or maxillary sinus) to the bifurcation of the rostral alveolar nerve. The diameter of the caudal part is relatively large as it may have carried the main trunk of the infraorbital nerve (Fig. 1B). It bears only two ramifications oriented ventrally toward two foramina for the innervation and nutrition of the upper lip. The caudal and rostral parts are supplemented by a third canal located more caudally which does not originate from the maxillary canal. These three canals may have borne the three alveolar rami of the CNV_2_ (Fig. 1B). The canal has a larger diameter at the level of the root of the rostral alveolar ramus, perhaps as a consequence of the anastomoses of the branches of the alveolar nerves, infraorbital nerve, and the palatine nerve as is the case in extant reptiles^13^,^14^. In extant mammals the infraorbital nerve (ION) is the branch of the CNV_2_ that diverges from the rostral alveolar nerve and passes through the infraorbital foramen^21^. In the absence of an infraorbital foramen in early cynodonts, the rostral-most part of the maxillary canal, that diverges from the rostral alveolar nerve, can be reasonably identified as the homologue of the tube for the ION (Fig. 1B, marked by the dotted line). This part of the maxillary canal houses the same ramifications of the ION as seen in extant mammals. Proximally, depending on the specimen, two to four ramifications are sent dorsally in dorso-caudal, dorsal, and dorso-rostral directions. These are identified as the external nasal (or palpebral) rami of the ION, even though they may have also innervated the skin covering the maxillary and frontal bones. The main branch of the maxillary canal tapers rostrally as it ramifies into numerous branches dorsally (internal nasal rami of the ION) and ventrally (superior labial rami of the ION).

The root of the maxillary canal is not ossified, such that the maxillary canal disappears caudally into the maxillary antrum, an air sinus. However, there is evidence for an early divergence of the caudal alveolar nerve because it does not originate from the maxillary canal, as in *Varanus* (Fig. 1A,B). This branch may have remained independent or may have joined the maxillary canal rostrally, because the bony tubes for the medial and rostral alveolar nerves originate from the maxillary canal (Fig. 1B).

The tube for the ophthalmic nerve is not ossified in *Cynosaurus* and *Galesaurus*. Only marks of the branches of the ophthalmic nerve are represented by some short bony channels that pierce the nasal and frontal bones. This is the condition in all NMT we have investigated, except *Thrinaxodon*. *Thrinaxodon* displays a complete ossification of the ducts for the ophthalmic nerve on the rostrum (Fig. 1C). This canal is divided into two main branches which are highly ramified themselves. There is a rostral branch to innervate the skin of the nasal bone (nasal ramus) and a caudal one to innervate the skin of the frontal bone (fontal ramus).

The cynognathians differ from the early cynodonts only by the less marked differences between the diameters of the stem of the maxillary canal and the ION. Also, the alveolar nerves do not originate from a side branch of the maxillary canal, which possibly means that they might have passed through the maxillary antrum (Fig. 1D).

Probainognathians are distinguished by the small number of external openings of the maxillary canal. *Ecteninion*, the basal-most genus of Probainognathia examined here, has only five visible foramina (Fig. 1E). Three foramina open on the extremity of the rostrum and the ION appears trifurcated. The dorsal and medial branches may have housed the internal nasal nerve, whereas the ventral-most branch might have housed the superior labial nerve. There is no evidence of an external nasal ramus of the ION. Caudally, only two of the three foramina for the alveolar rami are present. The caudal-most one is here identified as the foramen for the caudal alveolar ramus based on its independent origin in the maxillary antrum (Fig. 1E). The rostral foramen is connected to the maxillary canal, but it is not clear whether it is for the rostral or medial alveolar ramus (Fig. 1E). Unlike the more derived probainognathians, the course of the trigeminal nerve inside the maxillary antrum is not ossified, but a distinct foramen for the CNV_2_ is nevertheless present inside the orbit (Fig. 1E).

*Pachygenelus* and *Tritylodon* show the typical mammalian condition of the presence of a short infraorbital canal for the ION. As a consequence there is only one single and caudally retracted infraorbital foramen (Fig. 1F). In contrast to other NMT, the infraorbital canal is fully ossified, as in mammals. Three foramina are present rostral to the orbit, but only the infraorbital foramen transmitted the infraorbital nerve (Fig. 1F). The remaining two foramina, located more rostrally, originate through an independent canal from what Sues^22^ termed the sphenopalatine foramen (Fig. 1F). Given the pattern observed in the closely related *Ecteninion*, we choose here to homologize these foramina with those for the alveolar rami of the CNV_2_ (which implies that these canals would be homologous to the alveolar canals of extant mammals). However, they could also provide passage for the posterior superior lateral nasal rami or the nasopalatine rami of the CNV_2_. In any case, they do not appear to be related to the passage of the the infraorbital nerve.

**Figure 2:**
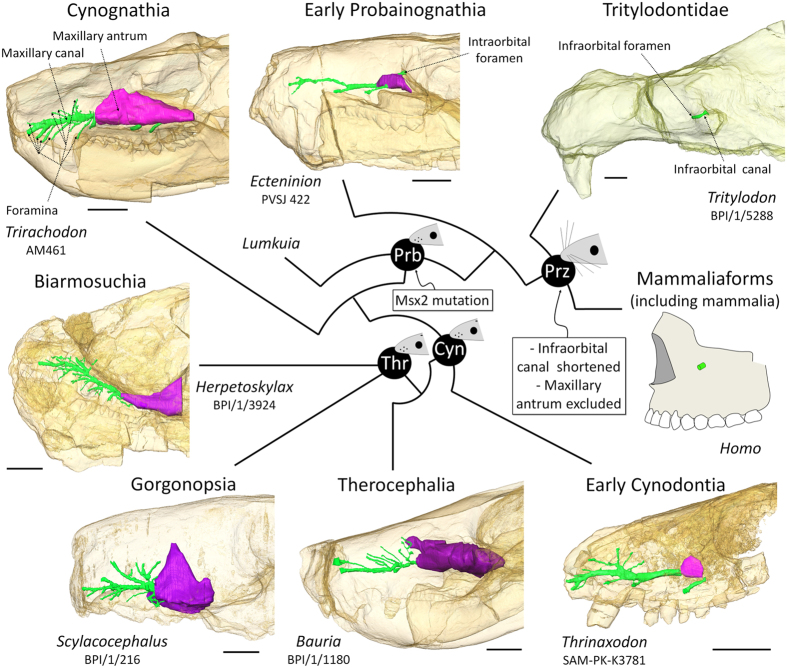
The evolution of the bony structures associated with the infraorbital nerve in Therapsida. Digital reconstruction of the maxillary canal (in green) and maxillary antrum (in purple) based on CT scan images (see Material and Methods). Scale bars = 10 mm. Phylogeny after references Rubidge and Sidor^31^ and Liu and Olsen^33^. Lateral views of the skulls with bones transparent. Rostral is to the left. Videos of the CT images of the specimens are available in the Supplementary Information. Abbreviations : Cyn, Cynodontia clade; Prb, Probainognathia clade; Prz, Prozonstrodontia clade; Thr, Therapsida clade.

In contrast to what is observed in early cynodonts, in therocephalians the caudal part of the maxillary canal for the main trunk of the infraorbital nerve appears shorter because it is not ossified (Fig. 1G). In *Bauria*, there appear to be five external nasal rami, all oriented dorso-rostrally (see Fig. 2).

In gorgonopsians, the lack of ossification of the caudal part of the maxillary canal is even more pronounced than in therocephalians. The maxillary canal is limited to the part corresponding to the infraorbital nerve. The caudal half of the canal is not ossified and is merged with the extensive maxillary antrum. There are only one or two distinct alveolar rami that open on the maxilla above the large canines (Fig. 1H), possibly as a result of the reduction of the post-canine dentition. In contrast, biarmosuchians show a condition similar to that in early cynodonts, except that all three alveolar rami originate from the maxillary canal (Fig. 1I). The caudal alveolar nerve is nevertheless connected to a side branch, which is reminiscent of the condition in early cynodonts (Fig. 1I).

## Discussion

### The passage of the infraorbital nerve and presence of maxillary vibrissae

In extant mammals and reptiles (non-avian sauropsids), facial sensitivity is relayed to the brain mainly by the maxillary branch of the trigeminal nerve, the so-called maxillary nerve (CNV_2_)^14^,^23^,^24^,^25^. This nerve (mainly its infraorbital branch, termed the maxillary nerve in non-mammals) passes, together with some small blood vessels and the palatine nerve (facial nerve), through the maxillary canal in the upper jaw^14^,^23^. In mammals, the maxillary canal is homologous to the infraorbital canal as it carries the infraorbital nerve and vessels^24^,^25^. The structure - sometimes termed the “maxillary canal” in mammals (but alveolar canal is usually preferred) - that carries nutrient vessels and the alveolar nerves to innervate teeth roots^21^ therefore does not correspond to the entire maxillary canal in therapsids, but only to a side branch related to the alveolar nerves (Fig. 1). Generally the infraorbital canal is short in mammals and extends rostrally under the orbit to the infraorbital foramen (Fig. 2), although the infraorbital canal can be secondarily quite long in monotremes^18^,^19^. In reptiles (non-avian sauropsids), the maxillary canal for the CNV_2_ is longer than in most mammals, ramifies, and then opens more rostrally into a number of foramina often aligned above the tooth row (Fig. 1A)^14^,^15^,^23^. Thus, the maxillary and infraorbital canals assume the same critical role in the innervation and nutrition of facial skin, and they can be used to provide direct evidence for the evolution of facial sensitivity in fossil taxa^25^,^26^,^27^. Based on extant phylogenetic braketing, CNV_2_ and other tissues supplying the facial skin of NMT likely passed through the maxillary canal. Accordingly, it has been proposed that some foramina located on the rostrum of NMT may have innervated the snout with large sensory maxillary vibrissae^16^,^27^,^28^,^29^. Such foramina and a ramified maxillary canal characterize most NMT^26^,^27^, which would imply a wide occurrence of maxillary vibrissae among mammaliaform progenitors (Fig. 2). However, because similar foramina are present in a number of lizards, snakes, and archosaurs^15^,^23^,^26^,^28^,^30^ (Fig. 1A), the only argument that remains in favour of the presence of maxillary vibrissae in NMT is their apparent close relationship to mammaliaforms^16^,^30^,^31^.

In mammals, the CNV_2_, after exiting the skull and traversing the ventral orbit, passes through the infraorbital canal and exits the canal by the infraorbital foramen at a position ventral to the rostral margin of the orbit. This part of the CNV_2_, the infraorbital nerve (ION), is the one responsible for facial and vibrissae sensitivity^24^,^25^. The mammalian face and cheeks are flexible, highly sensitive, and sometimes motile structures for whisking, implying that the branches of the ION must also be flexible to prevent damage during flexion to maintain innervation to the facial vibrissae. This facial flexibility is incompatible with the complete enclosure of CNV_2_ in a bony tube as seen in NMT^27^,^30^. As such, the infraorbital nerve no longer branches inside the maxilla in mammals, but rather in soft tissues of the face, which reflects the shorter and unbranched infraorbital canal observed in mammals compared to NMT (Figs 1 and 2).

The use of CT on some of the best preserved therapsid specimens from the extensive fossil record of the South African Karoo Supergroup enables us to reconstruct the transformation from a long, ramified, but not fully ossified maxillary canal to a short and retracted infraorbital canal for the ION in therapsids (Fig. 2). Non-mammaliaform Prozostrodontia (NMPZ) are considered the stem group of mammaliaforms^32^,^33^,^34^. In non-prozostrodontian therapsids, the maxillary canal is separated from the orbit by the maxillary antrum (or maxillary sinus)^16^,^17^,^35^, a condition not seen in Prozostrodontia (Fig. 2). As such, the pathway of the CNV_2_ from this antrum to the orbit might not have been ossified (Figs 1 and 2). *Ecteninion* and *Probainognathus*^36^, two basal probainognathians, appear intermediate between non-prozostrodontian therapsids and prozostrodontians for this condition as they display a partial ossification of the canal resulting in the presence of a foramen for the CNV_2_ inside the orbit (Fig. 1E).

In contrast, in the more derived Prozostrodontia (including mammaliaforms), the maxillary antrum no longer merges with the maxillary canal which is fully ossified (Figs 1 and 2). Serial grinding and CT scan analyses of the NMPZ *Oligokyphus*^37^, *Kayentatherium*^22^, *Brasilitherium*^38^, *Tritylodon*, and *Pachygenelus* (Figs 1 and 2) confirm that (i) the maxillary canal is fully ossified and forms a complete infraorbital canal, and (ii) that the infraorbital canal is retracted in NMPZ, as in mammals. The infraorbital foramen is accompanied by a variable number of lesser foramina in NMPZ, as in extant mammals and most Mesozoic mammaliaforms^39^,^40^, but the CT scan survey conducted here reveals that only the most proximal of these foramina may have carried the infraorbital nerve (Fig. 1). Similarly, CT data of *Brasilitherium*, the closest relative of mammaliaforms among NMT, show that the infraorbital foramen diverge immediatly under the rostral margin of the orbit while the other, more rostral foramina are supplied by a distinct canal (presumably for the alveolar nerves) that originate more caudally^38^, like in *Tritylodon* (Fig. 1). The poorly preserved maxillae attributed to the stem mammaliaform *Morganucodon* seems to display more than one large foramen^41^, but the CT scan of a weathered skull of *Morganucodon* available on Digimorph.org^12^,^36^ shows compelling evidence that the infraorbital canal is separated from the canal leading to these large foramina. This strongly suggests that the structures identified here as the infraorbital canal and foramen in NMPZ are truly homologous to the mammalian infraorbital canal and foramen. Only *Lumkuia* has numerous large foramina above the tooth row, suggesting branching of the proper maxillary canal, and matching its position as the basal-most Probainognathia^32^,^34^. *Ecteninion* and *Probainognathus* display a reduced number of openings with respect to *Lumkuia*^32^,^42^, which is more similar to the condition in NMPZ but still implies a ramified maxillary canal (Fig. 1E). In this respect, the presence of a short, unbranched infraorbital canal, similar in every way to the one providing passage to the ION for the vibrissae in therian mammals, strongly supports the existence of a flexible and sensitive face bearing tactile hairs in NMPZ (Fig. 2), some 240 mya^34^. This implies that NMPZ may already have possessed the genetic toolkit to produce hairs, including whiskers and by extension a fur coverage. This is consistent with Eckhart *et al*.^43^ who have already suggested that some genes for secreting the mammalian hard α-keratine may have been present in the last common ancestor of amniotes. Hence, the base material for hair was already available to NMPZ. The size of the infraorbital foramen in *Tritylodon* and *Pachygenelus* (Table 1) is similar to that in small mammals such as rodents which, given the strong correlation between IOF size and the vibrissae count^25^, strongly suggests that they may have displayed a similar number of macro- and microvibrissae. The presence in NMPZ of such facial tactile hairs would have aided orientation in the dark if these animals were nocturnal^8^,^9^,^10^.

### The parietal foramen, hair coverage and mammary glands

**Figure 3:**
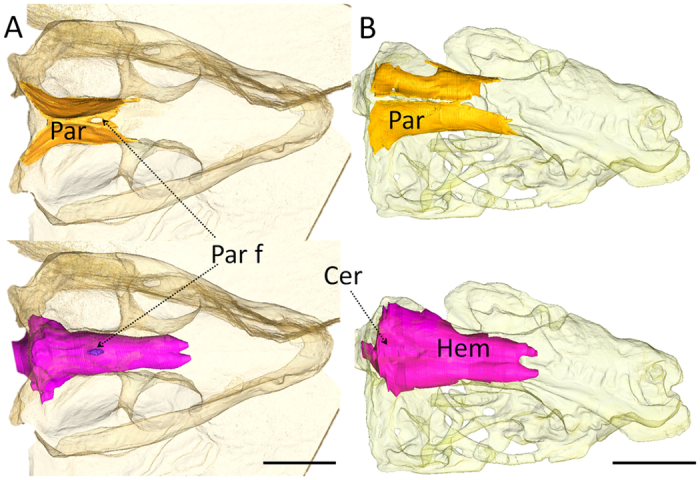
The skull roof of juvenile trirachodontid (A), BPI/1/4534 and juvenile *Pachygenelus* (B), BPI/1/5691 with the parietal bone (top, in yellow) and the cranial endocast reconstructed (below, in purple) based on CT scan images (see material and methods). Abbreviations: Cer, cerebellum; Hem: cerebral hemisphere; Par, parietal bone; Par f, parietal foramen.

Indirect evidence supporting the hypothesis of the evolution of hairs in Probainognathia comes from the fossil record of the parietal foramen, a midline opening located between the parietal bones (Fig. 3A). This foramen houses the third eye, or pineal eye, a photoreceptive organ that acts like a biological clock detecting daylight variation, for the modulation of biological rhythms and body temperature^44^,^45^. The parietal foramen was widespread in early vertebrates before being lost in most lineages, including synapsids^44^,^45^. Most NMT have a parietal foramen^31^,^40^,^44^,^45^, but occasional closure of the parietal foramen is documented, mostly in non-mammaliaform cynodonts (NMC) and in some therocephalians and dicynodonts^33^,^44^,^45^,^46^. The only therapsid group in which the parietal foramen is consistently absent is the Probainognathia, and it is now well established that the absence of a parietal foramen is a synapomorphy of the clade Probainognathia, including Mammaliaformes^31^,^39^,^40^,^45^.

In lizards, a parietal foramen forms during ontogeny when ossification of the fronto-parietal fontanel is interrupted in the region of the third eye^44^,^47^,^48^. Complete ossification of the fronto-parietal fontanel in these species is the result of the failure of the third eye to reach the skull roof^44^,^47^. In extant mammals, in which a parietal foramen is absent, the genetic pathway controlling the ossification of the fronto-parietal fontanel has been linked to the activity of the homeogene Msx2^4^,^49^,^50^,^51^. Indeed, Msx2 mutant humans and mice display a lack of ossification of the cranial roof. In Msx2 mutant humans this sometimes results in the persistence of a large circular opening on the midline between both parietal bones, reminiscent of the parietal foramen in NMT^52^. The activity of Msx2 has been linked to a number of developmental processes associated with the differentiation and proliferation of osteogenic cells, such as the morphogenesis and growth of the limbs^53^,^54^. In the skull, its expression is complemented by that of several other genes (e.g. Fgfr1, Twist) in the midsutural mesenchyme of the frontoparietal suture^50^, and is associated with the ossification of the posterior part of the cranial vault^51^. In a noteworthy manner, Msx2 has also been observed to have pleiotropic effects on the retina^55^ and the double knockout of Msx1 and Msx2 affects the ossification of the middle ear ossicles^56^. Despite this, it has been shown that the same mutation of Msx2, which leads to the persistence of the fronto-parietal foramen in mice, also causes deficiencies in hair follicle maintenance (except on the snout and around the orbits) and in the development of mammary glands^4^,^57^. These traits are prominent defining characteristics of extant mammals^31^,^33^,^40^. This coincidence suggests that the loss of the parietal foramen might be related to a mutation or changes in the expression or function of the Msx2 homeogene at the root of the Probainognathia clade, which would be consistent with the origin of hair in this group as hypothesized above (Fig. 2).

Msx2 mutant mice also fail to develop a large cerebellum, which demonstrates that this gene is also involved in the cerebellar development^4^. In contrast to other soft tissues, the fossil record of cerebellar size can be assessed using endocranial casts (physical or digital internal casts of the braincase). Among NMC, Probainognathia are the only species that display a clear enlargement of the cerebellum with distinct laterally expanded cerebellar hemispheres, as demonstrated previously^39^,^58^ (Fig. 3B), a feature not seen in other NMC. Taken together, these observations support the hypothesis that a mutation of the Msx2 homeogene may have occurred at the root of the Probainognathia clade in the Early Triassic, some 246 mya^34^. Many genes are involved in the mineralization of the bones of the cranial vault, and the development of the cerebellum, hairs, and mammary glands, but given that Msx2 plays a role in all these phenomena, it would be an extraordinary coincidence if all these characters did change at about the same time in the phylogenetic history of synapsids but were not accompanied by a mutation of Msx2. Finally, it must be noted that Msx2 plays a role in follicle maintenance, not in the development of hair^4^,^57^. Therefore, the existence of fur coverage could have predated this mutation at the root of the probainognathian clade, as suggested by the discovery of hair-like structures inside coprolites as early as the Late Permian^6^,^7^.

## Conclusion

*In silico* paleoneurology suggests that the evolution of hair occurred prior to the evolution of mammaliamorphs. First, fossilized evidence for the evolution of tactile facial hairs suggests that they might not have appeared prior to the reduction in the number of openings for the CNV_2_ and the appearance of a retracted infraorbital foramen in the NMPZ, at the root of the probainognathian clade at the very end of the Early Triassic, around 240–246 Ma (Fig. 2). Second, the complete disappearance of the parietal foramen in Probainognathia correlates with the definitive ossification of the fronto-parietal fontanelle in adult individuals, which, together with the enlargement of the cerebellum, suggests that the Msx2 gene mutated at the root of this clade (Fig. 2). This gene also pleiotropically controls hair follicle maintenance and the development of mammary glands, which implies that this mutation might have played a significant role in the evolution of both complete body hair coverage and lactation. Since the development of body hairs is believed to have increased body insulation^59^ and sensitivity^11^, this single mutation event might have been the root source of some of the most prominent characters defining mammals, such as hair coverage, lactation, lateral cerebellar expansion and endothermy. These typical mammalian characters might thus have first evolved in NMC.

## Material and Methods

Institutional abbreviations: AM: Albany Museum (Grahamstown, South Africa), BPI/1: Evolutionary Studies Institute (Johannesburg, South Africa); MS: School of Anatomical Science (Johannesburg, South Africa) SAM-PK: Iziko Museum (Cape Town, South Africa); PVSJ: Museo de Ciencias Naturales, Universidad Nacional de San Juan, Argentina; RC: Rubidge collection (Graaff-Reinet, South Africa); TM: Distsong (Transvaal) Museum (Pretoria, South Africa).

List of the scanned material:

Sauropsida

MS53: *Varanus* sp., voxel size: 0.0666 mm, dry skull of an extant species.

Biarmosuchia

BPI/1/816: Biarmosuchia, Burnetiamorph, *Lemurosaurus pricei*, voxel size: 0.05 mm, *Cistecephalus* AZ, Wuchiapigian, Late Permian.

BPI/1/3924: Biarmosuchia, Burnetiamorph, *Herpetoskylax hopsoni*, voxel size: 0.0689 mm, *Cistecephalus* AZ, Wuchiapigian, Late Permian.

Gorgonopsia

BPI/1/216: Gorgonopsia, *Scylacocephalus watermeyeri*, voxel size: 0.0589 mm, *Cistecephalus* assemblage zone (AZ), Wuchiapigian, Late Permian.

BPI/1/155: Gorgonopsia, indet. Gorgonopsia, voxel size: 0.0799 mm, *Cistecephalus* AZ, Wuchiapigian, Late Permian.

Therocephalia

BPI/1/100: Therocephalia, Whaitsiidae, *Theriognathus microps*, voxel size: 0.0801 mm, *Daptocephalus* AZ, Changhsingian, Late Permian.

BPI/1/512: Therocephalia, Whaitsiidae, *Theriognathus microps*, voxel size: 0.0756 mm, *Daptocephalus* AZ, Changhsingian, Late Permian.

BPI/1/1180: Therocephalia, Baurioidea, *Bauria cynops*, voxel size: 0.0668 mm, *Cynognathus* AZ, Anisian, Early Triassic.

BPI/1/3770: Therocephalia, Baurioidea, *Bauria cynops*, voxel size: 0.0728 mm, *Cynognathus* AZ, Anisian, Early Triassic.

BPI/1/3849: Therocephalia, Akidognathiidae, *Olivierosuchus parringtoni*, voxel size: 0.0655 mm, *Lystrosaurus* AZ, Induan, Early Triassic.

BPI/1/4401: Therocephalia, Hofmeyriidae, *Hofmeyria* sp., voxel size: 0.0555 mm, *Cistecephalus* AZ, Wuchiapigian, Late Permian.

Cynodontia

Early Cynodontia

BPI/1/3926: Cynodontia, *Cynosaurus suppostus*, voxel size: 0.0708 mm, *Daptocephalus* AZ, Changhsingian, Late Permian.

BPI/1/4469: Cynodontia, *Cynosaurus suppostus*, voxel size: 0.0342 mm, *Daptocephalus* AZ, Changhsingian, Late Permian.

BPI/1/1563: Cynodontia, *Cynosaurus suppostus*, voxel size: 0.0291 mm, *Daptocephalus* AZ, Changhsingian, Late Permian.

AM4947: Cynodontia, *Cynosaurus suppostus*, voxel size: 0.0476 mm, *Daptocephalus* AZ, Changhsingian, Late Permian.

BPI/1/7199: Cynodontia, *Thrinaxodon liorhinus*, voxel size: 0.03 mm, *Lystrosaurus* AZ, Induan, Early Triassic^60^.

SAM-PK-K3781: Cynodontia, *Thrinaxodon liorhinus*, voxel size: 0.0513 mm, *Lystrosaurus* AZ, Induan, Early Triassic.

BPI/1/4623: Cynodontia, *Thrinaxodon liorhinus*, voxel size: 0.050 mm, *Lystrosaurus* AZ, Induan, Early Triassic.

RC845: Cynodontia, *Galesaurus platyceps*, voxel size: 0.050 mm, *Lystrosaurus* AZ, Induan, Early Triassic.

TM80: Cynodontia, *Galesaurus platyceps*, voxel size: 0.050 mm, *Lystrosaurus* AZ, Induan, Early Triassic.

Cynodontia

Cynognathia

BPI/1/4534: Cynodontia, Cynognathia, indet.Trirachodontidae, voxel size: 0.0556 mm, *Cynognathus* AZ, Anisian, Early Triassic.

BPI/1/4658: Cynodontia, Cynognathia, *Trirachodon berryi*, voxel size: 0.0667 mm, *Cynognathus* AZ, Anisian, Early Triassic.

AM461: Cynodontia, Cynognathia, *Trirachodon berryi*, voxel size: 0.0668 mm, *Cynognathus* AZ, Anisian, Early Triassic.

BPI/1/5362: Cynodontia, Cynognathia, *Langbergia modisei*, voxel size: 0.0796 mm, *Cynognathus* AZ, Anisian, Early Triassic.

BPI/1/3776: Cynodontia, Cynognathia, *Diademodon tetragonus*, voxel size: 0.0801 mm, *Cynognathus* AZ, Anisian, Early Triassic.

Cynodontia

Probainognathia

BPI/1/5691: Cynodontia, Probainognathia, *Pachygenelus monus*, voxel size: 0.025 mm, *Massospondylus* range zone (RZ), Hettangian, Sinnemurian, and Pliensbachian, Early Jurassic.

BPI/1/4778: Cynodontia, Probainognathia, *Tritylodon longaevus*, voxel size: 0.0930 mm, *Massospondylus* RZ, Hettangian, Sinnemurian, and Pliensbachian, Early Jurassic.

BPI/1/5088: Cynodontia, Probainognathia, *Tritylodon longaevus*, voxel size: 0.0908 mm, *Massospondylus* RZ, Hettangian, Sinnemurian, and Pliensbachian, Early Jurassic.

BPI/1/5288: Cynodontia, Probainognathia, *Tritylodon* sp., voxel size: 0.0509 mm, *Massospondylus* RZ, Hettangian, Sinnemurian, and Pliensbachian, Early Jurassic.

PVSJ 422: *Ecteninion lunensis* after Digimorph.org, voxel size: 0.1624 mm^36^. Courtesy of Timothy Rowe of University of Texas, Department of Geological Sciences.

CT scanning: twenty nine therapsid skulls were scanned for this study. All specimens were scanned at the Evolutionary Studies Institute (ESI) scanning facility using *Nikon Metrology XTH 225/320 LC* except *Thrinaxodon* BPI/1/7199 that was scanned at the European Synchrotron Radiation Facility, Grenoble (see 60 for details) and *Ecteninion* which was scanned at the University of Texas (see the list of the scanned specimens for details). A list of specimens examined, with taxonomic assignment, voxel size, and stratigraphic position is provided above. Three-dimensional renderings of the internal structure of the maxillary canal, parietal bone, and cranial endocasts were obtained using manual segmentation under Avizo 8 (VSG). In order to focus our study on the bony structures that carried tissues actively playing a role in the innervation and nutrition of the face, and thus are relevant to the evolution of facial sensitivity and motility, only the parts of the canal that directly communicate with the external surface were segmented. For request concerning access to CT scan data, please contact K. Carlson (CT facility manager) : Kristian.Carlson@wits.ac.za. Area of the infraorbital foramen was obtained using direct measurement with a caliper.

## Additional Information

**Data availability**: For request concerning access to CT scan data, please contact K. Carlson (CT facility manager): Kristian.Carlson@wits.ac.za.

**How to cite this article**: Benoit, J. *et al*. Palaeoneurological clues to the evolution of defining mammalian soft tissue traits. *Sci. Rep*. **6**, 25604; doi: 10.1038/srep25604 (2016).

## Supplementary Material

Supplementary Information

## Figures and Tables

**Table 1:** Area of the infraorbital foramen (in mm^2^) in *Tritylodon* and *Pachygenelus*.

*Tritylodon*	Area of the infraorbital foramen
BPI/1/4778	7.32
BPI/1/5167	6.48
BPI/1/8089A	6.53
BPI/1/5289	7.03
BPI/1/4745	8.29
BPI/1/4265	6.88
BPI/1/5269	5.90
Average	6.92
*Pachygenelus*	
BPI/1/5691	3.75

## References

[b1] Martin T. . A Cretaceous eutriconodont and integument evolution in early mammals. Nature 526, 380–384 (2015). 26469049 10.1038/nature14905

[b2] Oftedal O. T. The mammary gland and its origin during synapsid evolution. J Mammary Gland Biol Neoplasia 7, 225–252 (2002). 12751889 10.1023/a:1022896515287

[b3] Lefèvre C. M., Sharp J. A. & Nicholas K. R. Evolution of Lactation: Ancient Origin and Extreme Adaptations of the Lactation System. Annu Rev Genomics Hum Genet 11, 219–38 (2010). 20565255 10.1146/annurev-genom-082509-141806

[b4] Satokata I. . Msx2 deficiency in mice causes pleiotropic defects in bone growth and ectodermal organ formation. Nat Genet 24, 391–395 (2000). 10742104 10.1038/74231

[b5] Ji Q., Luo Z.-X., Yuan C.-X. & Tabrum A. R. A swimming mammaliaform from the Middle Jurassic and ecomorphological diversification of early mammals. Science 311(5764), 1123–1127 (2006). 16497926 10.1126/science.1123026

[b6] Smith R. M. H. & Botha-Brink J. Morphology and composition of bone-bearing coprolites from the Late Permian Beaufort Group, Karoo Basin, South Africa. Palaeogeogr Palaeoclimatol Palaeoecol 312, 40–53 (2011).

[b7] Bajdek P. . Microbiota and food residues including possible evidence of pre-mammalian hair in Upper Permian coprolites from Russia. Lethaia 10.1111/let.12156 (2015).

[b8] Jerison H. J. Evolution of the brain and intelligence (Academic Press, New York, 1973).

[b9] Gerkema M. P. . The nocturnal bottleneck and the evolution of activity patterns in mammals. Proc R Soc B 280, 20130508 (2013). 10.1098/rspb.2013.0508 PMC3712437 23825205

[b10] Angielczyk K. D. & Schmitz L. Nocturnality in synapsids predates the origin of mammals by over 100 million years. Proc R Soc B 281, 20141642 (2014). 10.1098/rspb.2014.1642 PMC4173690 25186003

[b11] Rowe T. B. Coevolution of the mammalian middle ear and neocortex. Science 273, 651 (1996). 8662557 10.1126/science.273.5275.651

[b12] Rowe T. B., Macrini T. E. & Luo Z.-X. Fossil evidence on origin of the mammalian brain. Science 332, 955–957 (2011). 21596988 10.1126/science.1203117

[b13] Abdel-Kader T. G., Ali R. S. & Ibrahim N. M., The Cranial Nerves of *Mabuya quinquetaeniata* III: Nervus Trigeminus. Life Sci J 8, 650–669 (2011).

[b14] Bellairs A. D’. A. Observations on the snout of *Varanus*, and a comparison with that of other lizards and snakes. J Anat 83, 116–146 (1949). PMC1273152 18144962

[b15] Leitch D. B. & Catania K. C. Structure, innervation and response properties of integumentary sensory organs in crocodilians. J Exp Biol 215, 4217–4230 (2012). 23136155 10.1242/jeb.076836 PMC4074209

[b16] Fourie S. The cranial morphology of *Thrinaxodon liorhinus* Seeley. Ann S Af Mus 65, 337–400 (1974).

[b17] Sigurdsen T. New features of the snout and orbit of a therocephalian therapsid from South Africa. Acta Palaeontol Pol 51, 63–75 (2006).

[b18] Andres K. H., von During M., Iggo A. & Proske U. The anatomy and fine structure of the echidna *Tachyglossus aculeatus* snout with respect to its different trigeminal sensory receptors including the electroreceptors. Anat Embryol 184, 371–393 (1991). 1952110 10.1007/BF00957899

[b19] Manger P. R. & Pettigrew J. D. Ultrastructure, number, distribution and innervation of electroreceptors and mechanoreceptors in the bill skin of the platypus. Brain Behav Evol 48, 27–54 (1996). 8828862 10.1159/000113185

[b20] Rowe T. . The oldest platypus and its bearing on divergence timing of the platypus and echidna clades. Proc Natl Acad Sci USA 105(4), 1238–1242 (2008). 18216270 10.1073/pnas.0706385105 PMC2234122

[b21] Rodella L. F., Buffoli B., Labanca M. & Rezzani R. A review of the mandibular and maxillary nerve supplies and their clinical relevance. Arch Oral Biol 57(4), 323–334 (2012). 21996489 10.1016/j.archoralbio.2011.09.007

[b22] Sues H.-D. The skull and dentition of two tritylodontid synapsids from the Lower Jurassic of westem North America. Bull Mus Comp Zool 151, 217–268 (1986).

[b23] Düring von M. & Miller M. R. in Biology of the Reptilia Vol. 9 (eds Gans C., Northcutt R. G. & Ulinski P.), 407–411. (Academic Press, New York, 1979).

[b24] Muchlinski M. N. The relationship between the infraorbital foramen, infraorbital nerve, and maxillary mechanoreception: implications for interpreting the paleoecology of fossil mammals based on infraorbital foramen size. Anat Rec 291, 1221–1226 (2008). 10.1002/ar.20742 18780305

[b25] Muchlinski M. N. A comparative analysis of vibrissa count and infraorbital foramen area in primates and other mammals. J Hum Evol 58, 447–473 (2010). 20434193 10.1016/j.jhevol.2010.01.012

[b26] Tatarinov L. P. Morphological evolution of the Theriodonts and the general problems of Phylogenetics (NAUKA, Moscow, 1976).

[b27] Lingham-Soliar T. The Vertebrate Integument Volume 1 (Springer-Verlag: Berlin, Heidelberg, , 2014).

[b28] Van Valen L. Therapsids as mammals. Evolution 14, 304–313 (1960).

[b29] Watson D. M. S. On the skeleton of a bauriamorph reptile. J Zool 1931, 1163–1205 (1931).

[b30] Estes R. Cranial anatomy of the cynodont reptile *Thrinaxodon liorhinus*. Bull Mus Comp Zool 125, 165–180 (1961).

[b31] Rubidge B. S. & Sidor C. A. Evolutionary patterns among Permo-Triassic Therapsids. Annu Rev Ecol Syst 32, 449–480 (2001).

[b32] Hopson J. A. & Kitching J. W. A probainognathian cynodont from South Africa and the phylogeny of nonmammalian cynodonts. Bull Mus Comp Zool 156, 5–35 (2001).

[b33] Liu J. & Olsen P. The phylogenetic relationships of Eucynodontia (Amniota: Synapsida). J Mammal Evol 17, 151–176 (2010).

[b34] Ruta M., Botha-Brink J., Mitchell S. A. & Benton M. J. The radiation of cynodonts and the ground plan of mammalian morphological diversity. Proc Biol Sci 280, 20131865 (2014). 23986112 10.1098/rspb.2013.1865 PMC3768321

[b35] Kemp T. S. The Primitive Cynodont *Procynosuchus*: Functional Anatomy of the Skull and Relationships. Philos Trans R Soc Lond B Biol Sci 285, 73–122 (1979).

[b36] Rowe T. (Dir.)., Digimorph, Digital Morphology library (online), University of Texas Digital Morphology Group, http://digimorph.org (2002–2005) Date of access:06/09/2015.

[b37] Kuhne W. G. The Liassic therapsid Oligokyphus. (British Museum Natural History, London, 1956).

[b38] Ruf I., Maier W., Rodrigues P. G. & Schultz C. L. Nasal Anatomy of the Non-mammaliaform Cynodont *Brasilitherium riograndensis* (Eucynodontia, Therapsida) Reveals New Insight into Mammalian Evolution. Anat Rec 297, 2018–2030 (2014). 10.1002/ar.23022 25312362

[b39] Kielan-Jaworowska Z., Cifelli R. L. & Luo Z.-X. Mammals from the Age of Dinosaurs Origins, Evolution, and Structure (Columbia University Press, New York, 2004).

[b40] Kemp T. S. The origin and evolution of mammals (Oxford University Press, Oxford, 2005).

[b41] Kermack K. A., Mussett F. & Rigney H. W. The skull of *Morganucodon*. Zool J Linn Soc 71, 1–158 (1981).

[b42] Martinez R. N., May C. L. & Forster C. A. A new carnivorous cynodont from the Ichigualasto Formation (Late Triassic, Argentina), with comments on eucynodont phylogeny. J Vert Paleontol 16, 271–284 (1996).

[b43] Eckhart L. . Identifcation of reptilian genes encoding hair keratin-like proteins suggests a new scenario for the evolutionary origin of hair. Proc Natl Acad Sci USA 105, 18419–18423 (2008). 19001262 10.1073/pnas.0805154105 PMC2587626

[b44] Quay W. B. In Biology of the Reptilia Vol. 9 (eds Gans C., Northcutt R. G. & Ulinski P.), 245–406 (Academic Press, New York, 1979).

[b45] Benoit J., Abdala F., Manger P. R. & Rubidge B. S. The sixth sense in mammals forerunners: variability of the parietal foramen in South African Therapsida suggests convergent evolution of their physiology. Acta Paleontol Pol, doi: 10.4202/app.00219.2015 (2016).

[b46] Benoit J. . Physiological implications of the abnormal absence of the parietal foramen in a late Permian cynodont (Therapsida). Naturwissenschaften 102(11), 69, doi: 10.1007/s00114-015-1321-4 (2015). 26538062

[b47] Roth J. J., Roth E. C. & Hotton N. N. In Ecology and Biology of Mammal-like Reptiles (eds Hotton N., MacLean P. D., Roth J. J., Roth E. D.), 173–184 (Smithsonian Institution Press, Washington, 1986).

[b48] de Beer G. R. The Development of the Vertebrate Skull (Oxford University Press, 1937).

[b49] Garcia-Miñaur S. ., Parietal foramina with cleidocranial dysplasia is caused by mutation in MSX2. Eur J Hum Genet 11, 892–895 (2003). 14571277 10.1038/sj.ejhg.5201062

[b50] Morriss-Kay G. M. Derivation of the mammalian skull vault. J Anat 199, 143–151 (2001). 11523816 10.1046/j.1469-7580.2001.19910143.x PMC1594961

[b51] Roybal P. G. . Inactivation of Msx1 and Msx2 in neural crest reveals an unexpected role in suppressing heterotopic bone formation in the head. Dev Biol. 343, 28–39 (2010). 20398647 10.1016/j.ydbio.2010.04.007 PMC3279331

[b52] Agarwal P., Pandey M., Baranwal S. & Roy K., Large midline persistent parietal foramina with occipital encephalocele and abnormal venous drainage. J Cleft Lip Palate Craniofac Anomal 2, 66–9 (2015).

[b53] Akimenko M. A., Johnson S. L., Westerfield M. & Ekker M. Differential induction of four msx homeobox genes during fin development and regeneration in zebrafish. Development 121, 347–357 (1995). 7768177 10.1242/dev.121.2.347

[b54] Tokita M., Chaeychomsri W. & Siruntawineti J. Skeletal gene expression in the temporal region of the reptilian embryos: implications for the evolution of reptilian skull morphology. SpringerPlus 2, 336–356 (2013). 24711977 10.1186/2193-1801-2-336 PMC3970585

[b55] Lamba D. A. & Reh T. A. Microarray Characterization of Human Embryonic Stem Cell–Derived Retinal Cultures. Invest Ophthalmol Vis Sci 52, 4897–4906 (2011). 21345990 10.1167/iovs.10-6504 PMC3175935

[b56] Zhang Z. . Malleal processus brevis is dispensable for normal hearing in mice. Dev Dyn 227, 69–77 (2003). 12701100 10.1002/dvdy.10288

[b57] Ferguson M. W. J. A hole in the head. Nat Genet 24, 330–331 (2000). 10742087 10.1038/74132

[b58] Hopson J. A. In Biology of the Reptilia (eds Glans C., Northcutt R. G., Ulinski P.), 39–146 (Academic Press, New York, 1979).

[b59] Hillenius W. J. & Ruben J. A. The Evolution of Endothermy in Terrestrial Vertebrates: Who? When? Why? Physiol Biochem Zool 77, 1019–1042 (2014). 15674773 10.1086/425185

[b60] Fernandez V. . Synchrotron Reveals Early Triassic Odd Couple:Injured Amphibian and Aestivating Therapsid Share Burrow. PLoS One 8(6), e64978 (2013). 23805181 10.1371/journal.pone.0064978 PMC3689844

