# Supplementary material for: Palaeoneurological clues to the evolution of defining mammalian soft tissue traits
Source: Sci Rep. 2016 May 9;6:25604. doi: 10.1038/srep25604 (PMC4860582; doi:10.1038/srep25604)
Supplement: Supplementary Information [file srep25604-s1.pdf]

Palaeoneurological clues to the evolution of defining mammalian soft tissue traits

Authors: J. Benoit[1,2*], P.R. Manger[2], B.S. Rubidge[1,3]

[1]Evolutionary Studies Institute (ESI), University of the Witwatersrand, PO Wits, 2050, Johannesburg, South Africa

[2]School of Anatomical Sciences, University of the Witwatersrand, 7 York Road, Parktown, 2193, Johannesburg, South Africa.

[3]School for Geosciences, University of the Witwatersrand, PO Wits, 2050, Johannesburg, South Africa

Key words: Msx2, Parietal foramen, Maxillary nerve, Hair, Therapsida

**Corresponding Author*: Julien Benoit – julien.benoit@wits.ac.za

Supplementary information

Videos of the CT images of the specimens illustrated in the manuscript. The maxillary canal is highlighted in green and the maxillary antrum is highlighted in purple. The same videos in high resolution are available at :

https://drive.google.com/open?id=0BwhRMMJx6StKSWhaNURWVGpuNFE
